# Exposure-relevant microplastic characteristics in drinking water and estuarine environments: environmental parameters for musculoskeletal experimental research

**DOI:** 10.3389/fpubh.2026.1926761

**Published:** 2026-08-26

**Authors:** Muhammad Adil Malik, Song Wu, Wenxiu Zhang, Junjie Huang, Xu Cao, Muhammad Salman Azhar

**Affiliations:** The Third Xiangya Hospital, Central South University, Changsha, China

**Keywords:** drinking water, environmental characterization, estuarine systems, microplastics, musculoskeletal research, particle morphology, particle size, polymer composition

## Abstract

**Background:**

Microplastics are heterogeneous environmental contaminants whose transport, persistence, and potential biological interactions may depend on particle morphology, size, geometry, and polymer composition. Environmental monitoring studies commonly describe particle occurrence within individual matrices, whereas experimental studies frequently use standardized particles that may not adequately represent environmentally observed configurations.

**Objective:**

This study aimed to characterize microplastic morphology, dimensional characteristics, size distribution, and polymer composition in drinking-water and estuarine datasets and to identify environmentally observed particle profiles that may inform future musculoskeletal research.

**Methods:**

Two publicly available environmental datasets were analyzed separately. Overall morphology was summarized across 44,221 San Francisco Bay particle records with valid classifications. For matrix-level analyses, quality-assurance samples were excluded, and sample-level morphology proportions were compared using permutational multivariate analysis of variance based on Bray–Curtis dissimilarities with 9,999 permutations. Homogeneity of multivariate dispersion was assessed using PERMDISP. Particle length and aspect ratio were analyzed using generalized estimating equations accounting for particles clustered within samples and adjusted for environmental matrix. Drinking-water records were analyzed descriptively because of heterogeneity in observational units and analytical methods.

**Results:**

In the complete archived particle inventory, fibers were the most frequently recorded morphology (49.8%, *n* = 22,012), followed by fragments (40.3%, *n* = 17,804). Among 266 environmental or biological samples retained for inferential analysis after exclusion of quality-assurance samples, sample-level morphology profiles differed significantly among matrices (PERMANOVA pseudo-*F* = 34.14, R2 = 0.344, *p* < 0.001), although multivariate dispersion also differed (PERMDISP *F* = 94.31, p < 0.001). In cluster-adjusted models, fibers were approximately 2.51 times longer than fragments (95% CI, 2.35–2.68) and had 24.76 times their aspect ratio (95% CI, 22.70–27.01).

**Conclusion:**

Environmental microplastics occurred as heterogeneous combinations of fibers, fragments, particle sizes, and polymer categories. These environmentally observed combinations of morphology, dimensions, and material identity may provide empirically grounded parameters for selecting more representative particles in future cartilage-, synovium-, and bone-related experimental studies. However, this study does not measure human intake, internal exposure, tissue accumulation, biological responses, or musculoskeletal outcomes and therefore does not establish toxicological risk, clinical association, or causality.

## 1 Introduction

Microplastics (MPs), defined as synthetic polymer particles smaller than 5 mm, have emerged as persistent environmental contaminants detected across aquatic, terrestrial, and atmospheric environments. The continuous expansion of plastic production, inadequate waste management, and fragmentation of larger plastic materials have resulted in widespread environmental accumulation of MPs. Aquatic systems, particularly freshwater environments, estuaries, and coastal regions, represent important reservoirs because they integrate inputs from wastewater discharge, urban runoff, atmospheric deposition, and plastic degradation processes. Human exposure to MPs may occur through multiple pathways, including ingestion of contaminated food and drinking water and inhalation of airborne particles. However, environmental microplastic contamination is highly heterogeneous, and the particle characteristics that influence persistence, transport behavior, and potential biological interactions remain incompletely understood (1–3).

Microplastics are not a uniform class of environmental pollutants; rather, their biological and ecological relevance may depend on specific physicochemical characteristics, including particle size, morphology, aspect ratio, surface properties, and polymer composition. Morphological characteristics are particularly important because particle geometry may influence environmental transport, degradation behavior, cellular interaction, and biological responses. Experimental studies have demonstrated that elongated fibers and irregular fragments may interact differently with biological systems compared with spherical particles, potentially affecting cellular uptake, inflammatory responses, oxidative stress, and clearance mechanisms. Therefore, detailed characterization of environmental microplastic morphology and size distribution represents an essential step toward understanding exposure scenarios and improving the biological relevance of environmental monitoring studies (4–6).

Emerging experimental evidence has raised interest in the potential effects of microplastics on musculoskeletal tissues, including cartilage, synovium, and bone. However, the environmental relevance of many existing experimental models remains uncertain because they commonly employ standardized spherical particles with predefined polymers and size ranges, whereas environmentally occurring microplastics are heterogeneous in morphology, dimensions, and material composition. Characterizing these environmental features is therefore necessary to define more representative exposure conditions for future musculoskeletal experiments. The musculoskeletal rationale for the present study lies in this environmental-to-experimental translation rather than in demonstrating toxicity, clinical association, or disease causation (7–10).

Despite growing attention toward the potential health implications of microplastics, an important knowledge gap remains between environmental exposure characterization and disease-oriented mechanistic research. Existing environmental studies have largely focused on the occurrence, abundance, and distribution of microplastics, whereas the detailed characterization of exposure-relevant particle features—including morphology, polymer identity, and size distribution—across different environmental compartments remains limited. Establishing environmental particle profiles is essential because these characteristics may determine which particle types are most relevant for future toxicological studies, biomonitoring strategies, and epidemiological investigations. In this context, environmental characterization can provide an important upstream framework for selecting biologically relevant microplastic exposure conditions in studies investigating potential relationships with diseases such as OA (5, 6, 11).

**Figure 1:**
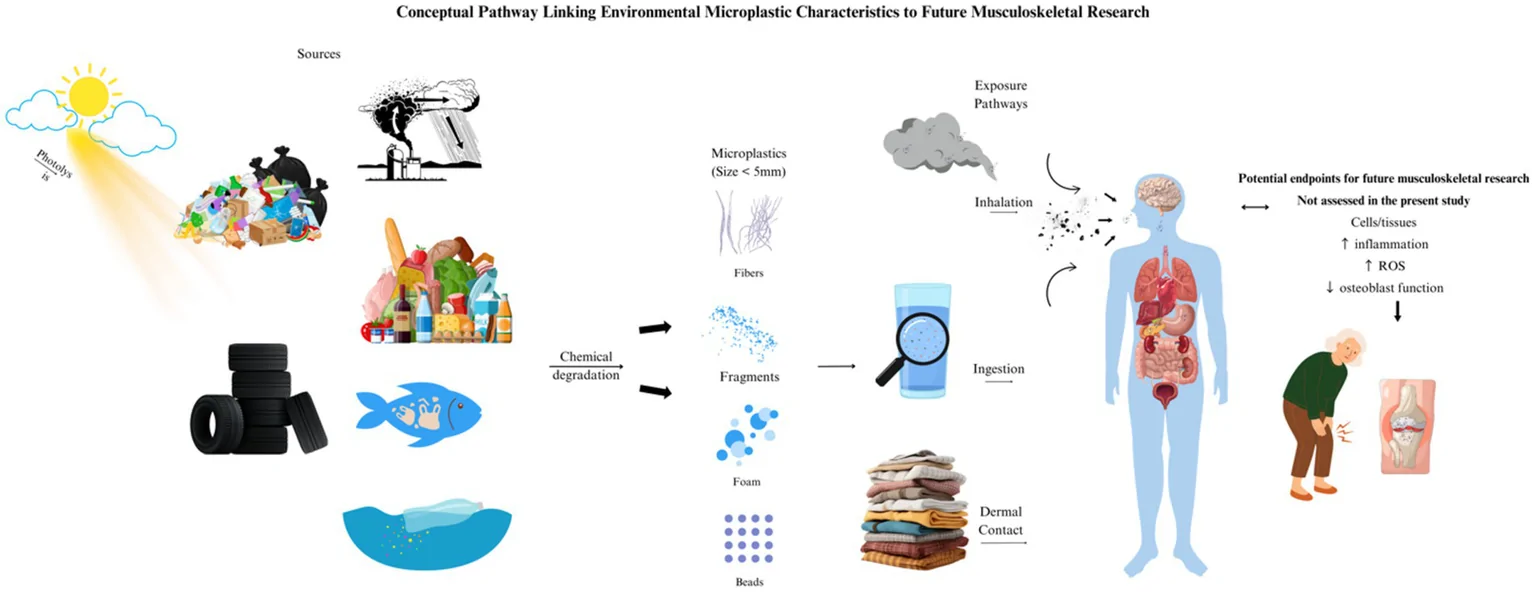
Conceptual pathway linking environmental sources, exposure routes, and microplastic characteristics with parameters relevant to future musculoskeletal experimental research. Human exposure, biological responses, and musculoskeletal outcomes were not assessed in the present study.

Accordingly, this study aimed to characterize microplastic morphology, dimensional characteristics, size distribution, and material composition using publicly available drinking-water and estuarine datasets. The primary environmental objective was to quantify these characteristics while preserving the original observational structure of each dataset. The translational objective was to identify environmentally observed combinations of particle shape, dimensions, and material identity that may inform the selection of more representative particles for future cartilage-, synovium-, and bone-related experiments. The study was not designed to estimate human exposure, tissue accumulation, toxicological risk, clinical association, or musculoskeletal disease causation. The environmental-to-experimental framework underlying the study is illustrated in Figure 1.

## 2 Materials and methods

### 2.1 Data sources

This study was a secondary analysis of two publicly available environmental microplastic datasets with different observational structures.

The primary particle-level dataset was obtained from the San Francisco Bay Microplastics Project, developed by the San Francisco Estuary Institute and 5 Gyres Institute and hosted through the California Natural Resources Agency Open Data Portal. The inventory contained 44,226 particle-level records collected across multiple environmental matrices and included information on morphology, colour, particle dimensions, and polymer or material identity where available. Five records without valid morphological classifications were excluded from morphology-based analyses, resulting in an analytical population of 44,221 particles.

The second data source was the publicly available Microplastics in Drinking Water dataset, published through the California Open Data Portal by the California State Water Resources Control Board. The dataset was developed through extraction and harmonization of data from peer-reviewed studies of bottled and tap drinking water and was accompanied by sample-validation and methodology files. The present analysis used the wide-format sample file (samples_geocoded.csv) and the associated methodology file (sample_methodology.csv). Records included information on drinking-water source, particle morphology, reported size categories, polymer composition, and analytical methodology where available. One row represented a study-specific sample or record-level observation rather than an individual particle. Because the source studies used heterogeneous sampling and analytical procedures, the drinking-water data were analyzed descriptively and were not quantitatively pooled with the particle-level San Francisco Bay inventory. Because the San Francisco Bay inventory was structured at the particle level, whereas the drinking-water compilation was structured at the sample or record level, the two datasets were analyzed separately and were not quantitatively pooled. No new environmental sampling or laboratory measurements were conducted. The complete archived inventory was retained for descriptive reporting of overall morphology frequencies. For inferential analyses, samples classified in the original metadata as field blanks, laboratory blanks, or laboratory quality-assurance samples were excluded. Accordingly, PERMANOVA, PERMDISP, and generalized estimating-equation analyses were restricted to environmental or biological samples.

### 2.2 Particle characterization and data quality

Morphological categories were retained according to the source dataset and comprised fiber, fragment, foam, film, sphere, and fiber bundle. Morphology-based analyses included records with a valid morphological classification.

Particle length and width were evaluated where available. Dimensional information was not available for every particle and missing values were not imputed. Microplastic-specific length analyses were restricted to records satisfying:


0<length≤5mm.

Records with missing, zero, negative, or greater-than-5-mm length values were excluded from microplastic-specific dimensional summaries. Extreme values were not automatically rescaled or recoded without verifiable source documentation.

Aspect ratio was calculated as:


Aspect ratio=particle length/particle width

Aspect-ratio analysis included only records with positive length and width values, particle width not exceeding particle length, and particle length no greater than 5 mm.

Polymer or material categories were retained according to the original source classifications. Named polymers, generic anthropogenic assignments, unknown materials, and uncharacterized records were maintained as separate categories. Polymer distributions were interpreted only within records with an available assignment and were not extrapolated to particles lacking chemical characterization.

### 2.3 Statistical analysis

Data processing, statistical analysis, and visualization were performed using R version *2025.09.0 + 387*, with the vegan and geepack packages. Categorical variables were summarized as frequencies and percentages, and continuous variables as medians with interquartile ranges because particle dimensions were right-skewed.

For matrix-level analysis, counts of the six morphological categories were aggregated by unique sample and converted to within-sample proportions. Differences in sample-level morphological composition among environmental matrices were evaluated using permutational multivariate analysis of variance based on Bray–Curtis dissimilarities with 9,999 permutations. Homogeneity of multivariate dispersion was assessed using permutation testing of distances to group centroids.

Particle length and aspect ratio were natural-log transformed and analyzed using Gaussian generalized estimating equations with an exchangeable working-correlation structure, sample identifier as the clustering variable, robust standard errors, and adjustment for environmental matrix. Missing values were not imputed, and the number of records contributing to each analysis was reported separately. The drinking-water compilation and material-assigned records were summarized descriptively because of heterogeneity in the original analytical methods and sparsity across material categories. All tests were two-sided, with *p* < 0.05 considered statistically significant.

### 2.4 Material-characterized subset

Material information was summarized for all records with an available assignment. Original material categories were retained and were distinguished from particles reported as unknown, uncharacterized, or broadly anthropogenic. Because material characterization was incomplete and may have followed source-specific subsampling procedures, material findings were interpreted conditionally within the characterized subset and were not extrapolated to the complete particle inventory.

## 3 Results

### 3.1 Dataset characteristics and morphological distribution

The San Francisco Bay Microplastics Project inventory contained 44,226 particle-level records. Five records without valid morphological classifications were excluded, leaving 44,221 particles for the full-inventory morphology analysis.

**Figure 2:**
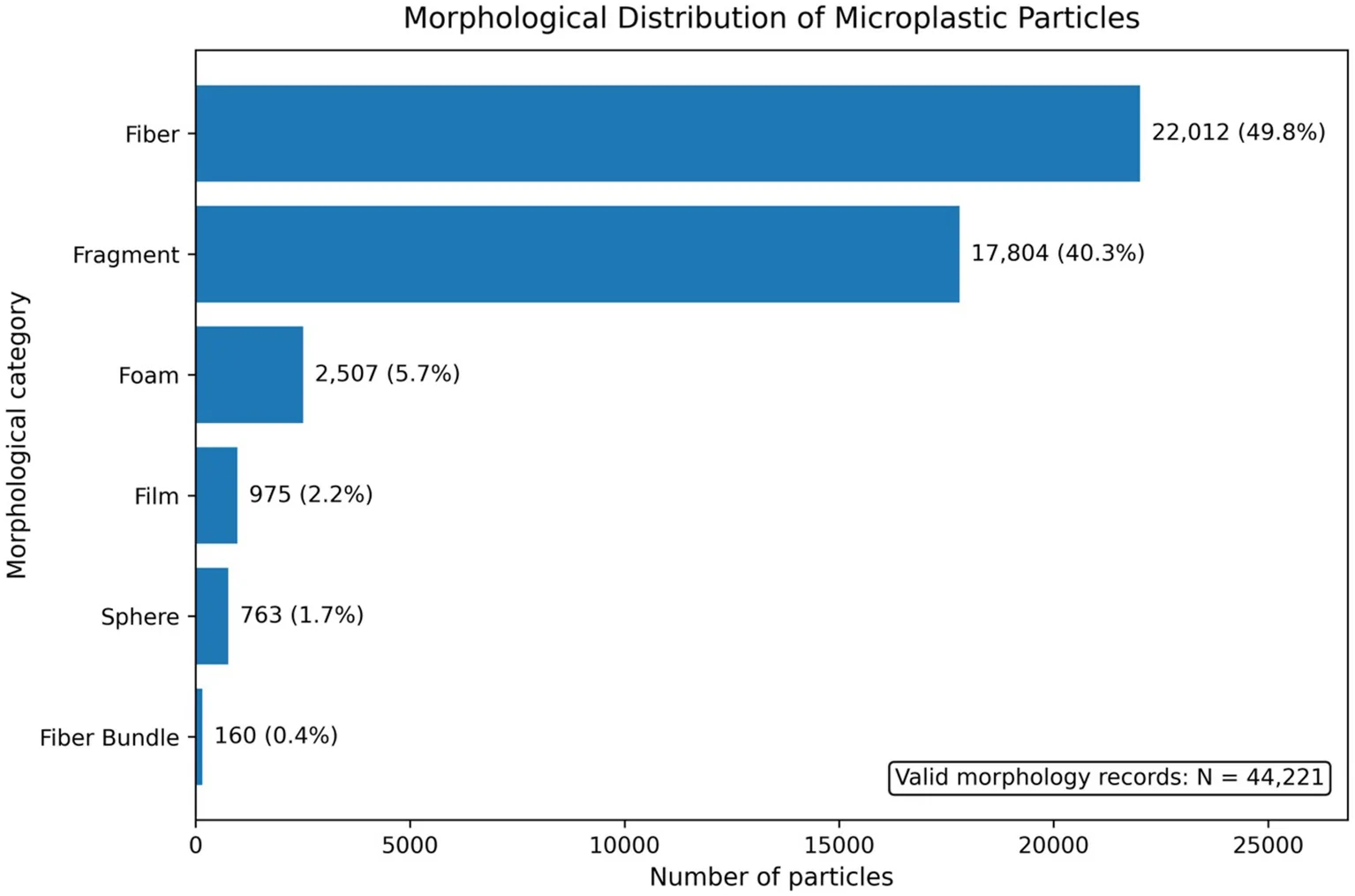
Morphological distribution of microplastics in the complete archived San Francisco Bay particle inventory. Percentages were calculated among 44,221 records with valid morphological classifications.

Fibers were the most frequently recorded category (49.8%, *n* = 22,012), followed by fragments (40.3%, *n* = 17,804). Foam accounted for 5.7% (*n* = 2,507), film for 2.2% (*n* = 975), spheres for 1.7% (*n* = 763), and fiber bundles for 0.4% (*n* = 160). Fibers and fragments together accounted for approximately 90% of particles with valid morphological classifications (Figure 2).

For inferential analysis, 40 quality-assurance samples were excluded, leaving 266 environmental or biological samples. Sample-level morphology profiles differed significantly among matrices (PERMANOVA pseudo-*F* = 34.14, *R*^2^ = 0.344, *p* < 0.001). Multivariate dispersion also differed among matrices (PERMDISP *F* = 94.31, *p* < 0.001). The result was therefore interpreted as overall matrix-specific heterogeneity reflecting differences in both average morphology profiles and within-matrix variability.

### 3.2 Particle size and geometric characteristics

After application of the prespecified dimensional eligibility criteria and exclusion of quality-assurance samples, 18,102 particles across 264 sample clusters were included in the particle-length analysis. Generalized estimating-equation models were adjusted for environmental matrix and accounted for clustering of particles within samples.

Relative to fragments, fibers were approximately 2.51 times longer on the geometric-mean scale (geometric-mean ratio, 2.51; 95% CI, 2.35–2.68; *p* < 0.001). Films (geometric-mean ratio, 1.23; 95% CI, 1.09–1.39; *p* = 0.001) and fiber bundles (geometric-mean ratio, 1.71; 95% CI, 1.46–2.00; *p* < 0.001) were also longer than fragments. Spheres were shorter than fragments (geometric-mean ratio, 0.58; 95% CI, 0.51–0.67; *p* < 0.001), whereas foam did not differ significantly from fragments (geometric-mean ratio, 1.10; 95% CI, 0.94–1.29; *p* = 0.244).

Aspect-ratio eligibility criteria were satisfied by 18,013 particles across 264 sample clusters. Fibers had an estimated aspect ratio 24.76 times that of fragments on the geometric-mean scale (95% CI, 22.70–27.01; *p* < 0.001). Fiber bundles also had higher aspect ratios than fragments (geometric-mean ratio, 2.20; 95% CI, 1.33–3.64; *p* = 0.002). Foam (geometric-mean ratio, 0.72; 95% CI, 0.64–0.80; *p* < 0.001) and spheres (geometric-mean ratio, 0.49; 95% CI, 0.43–0.55; *p* < 0.001) had lower aspect ratios than fragments. Film and fragment aspect ratios did not differ significantly (geometric-mean ratio, 0.97; 95% CI, 0.86–1.10; *p* = 0.604). Complete model estimates are presented in Table 1.

**Table 1:** Generalized estimating-equation analyses of particle length and aspect ratio.

Outcome	Comparison with fragments	Geometric-mean ratio	95% CI	*p* value
Length	Fiber	2.51	2.35–2.68	<0.001
Length	Foam	1.10	0.94–1.29	0.244
Length	Film	1.23	1.09–1.39	0.001
Length	Sphere	0.58	0.51–0.67	<0.001
Length	Fiber bundle	1.71	1.46–2.00	<0.001
Aspect ratio	Fiber	24.76	22.70–27.01	<0.001
Aspect ratio	Foam	0.72	0.64–0.80	<0.001
Aspect ratio	Film	0.97	0.86–1.10	0.604
Aspect ratio	Sphere	0.49	0.43–0.55	<0.001
Aspect ratio	Fiber bundle	2.20	1.33–3.64	0.002

Models were fitted to natural-log-transformed outcomes using Gaussian generalized estimating equations with an exchangeable working-correlation structure and robust standard errors. Sample identifier was the clustering variable. Models were adjusted for environmental matrix. Values greater than 1 indicate greater length or aspect ratio than fragments.

### 3.3 Microplastic characteristics in drinking-water datasets

Analysis of drinking-water records demonstrated frequent detection of smaller microplastic particles, particularly within submillimeter size categories. Particles below 500 μm represented a substantial proportion of reported size distributions across available records.

### 3.4 Material composition and morphology

Material labels other than “Not Characterized” were available for 7,119 particle records. Material–morphology patterns were summarized descriptively because material characterization was incomplete, category frequencies were highly imbalanced, and several material categories contained relatively few observations.

Polyester was predominantly recorded as fibers (89.8%), with the remaining polyester records classified as fiber bundles. Polyethylene was most frequently recorded as fragments (53.8%), followed by spheres (26.1%) and films (15.8%). Polypropylene was also predominantly represented by fragments (63.1%), whereas polystyrene was most frequently associated with foam morphology (65.9%). These patterns indicate that polymer or material identity and particle morphology should be considered jointly when selecting environmentally representative particles. They were not extrapolated to records lacking material characterization.

## 4 Discussion

### 4.1 Environmental microplastic profiles and morphological characteristics

In this secondary analysis of publicly available environmental datasets, we characterized microplastic profiles across drinking-water and estuarine systems, with particular emphasis on morphology, particle size, and polymer composition. The large particle-level inventory demonstrated that fibers and fragments represented the dominant morphological categories, together accounting for approximately 90% of classified particles. This observation is consistent with previous studies showing that fibrous and fragmented microplastics are among the most frequently detected morphologies in aquatic environments (1, 2, 6).

The predominance of fibers likely reflects the widespread contribution of synthetic textile materials, wastewater discharge, and urban runoff to aquatic microplastic contamination. Synthetic fibers derived from clothing and industrial materials have been repeatedly identified as major contributors to environmental microplastic burdens because they can be continuously released during textile production, washing, and degradation processes (12, 13). Fragment-dominated profiles, meanwhile, are generally associated with the progressive breakdown of larger plastic products, including packaging materials and consumer plastics (2, 14). Together, the observed dominance of fibers and fragments highlights the importance of considering particle morphology as a key component of environmental microplastic characterization.

Particle geometry may influence environmental transport, persistence, and potential interactions with biological systems. Fibrous particles exhibit distinct physical properties compared with more compact fragments, including greater aspect ratios and different surface-to-volume relationships. Previous experimental studies have demonstrated that particle morphology can influence cellular uptake, clearance mechanisms, and inflammatory responses, suggesting that morphology represents an important parameter beyond simple particle abundance measurements (4, 15). Therefore, environmental monitoring approaches incorporating morphology and dimensional characteristics may provide more biologically informative exposure profiles.

### 4.2 Exposure pathways and relevance of particle characteristics

The detection of small-sized particles in drinking-water datasets provides additional context regarding potential exposure pathways. Smaller microplastic particles may exhibit increased environmental mobility and may be more readily transported between environmental compartments compared with larger particles (1, 2). Aquatic systems also represent important interfaces for trophic transfer because microplastics can be ingested by aquatic organisms and subsequently move through food webs (11).

However, exposure interpretation requires careful consideration of environmental concentration, particle characteristics, analytical methodology, and biological context. The presence of environmentally detected particles does not directly indicate internal human exposure or biological effects. Current evidence suggests that human exposure to microplastics occurs through multiple pathways, including food, water, and air, but the magnitude of exposure and the long-term health implications remain incompletely defined (1, 16).

In this context, the value of environmental characterization studies lies in defining the particle profiles to which organisms and humans may be exposed. The morphology, size distribution, and polymer composition identified in this study may provide relevant parameters for future experimental designs investigating environmentally realistic microplastic exposures.

### 4.3 Implications for environmentally representative musculoskeletal experimental models

The principal musculoskeletal relevance of the present analysis lies in experimental design rather than in direct evidence of biological or clinical effects. Many microplastic toxicology studies use standardized spherical polymer particles, particularly polystyrene beads, because they are experimentally convenient and commercially available. However, the present environmental inventory was dominated by fibers and fragments, which together accounted for approximately 90% of particles with valid morphological classifications. This discrepancy indicates that experimental models relying exclusively on spherical particles may not fully represent the geometric heterogeneity observed in environmental systems (17).

The dimensional analyses further demonstrated that morphology was not merely a descriptive label. After adjustment for environmental matrix and within-sample clustering, fibers were approximately 2.51 times longer than fragments and had approximately 24.76 times their aspect ratio. These substantial geometric differences may affect particle–cell contact, sedimentation, uptake, clearance, and surface interactions. Future musculoskeletal experiments should therefore consider morphology as an independent experimental variable and compare elongated fibers, irregular fragments, and standardized spherical particles under otherwise controlled conditions. Relevant models may include chondrocytes, synoviocytes, osteoblasts, osteoclasts, macrophages, and tissue-engineered cartilage or bone systems (5, 7, 8). Material identity should also be considered jointly with particle morphology. Within the material-characterized subset, polyester was predominantly recorded as fibers, whereas polyethylene and polypropylene were more frequently represented by fragments. Consequently, an experiment comparing polymers without controlling for morphology could conflate material-related effects with geometry-related effects. Environmentally representative musculoskeletal studies should therefore report and control particle morphology, dimensions, polymer identity, particle number, mass concentration, and chemical-confirmation procedures (17).

These recommendations remain hypothesis-generating. The present study did not assess particle uptake, tissue accumulation, inflammatory signaling, cartilage degradation, bone remodeling, or clinical outcomes. Its contribution is to identify environmentally documented particle profiles that can guide the design of future biological investigations rather than to establish musculoskeletal toxicity or disease risk (4, 15). The implications of the principal environmental findings for future musculoskeletal experimental design are summarized in Table 2.

**Table 2:** Environmental microplastic findings and implications for future musculoskeletal experimental design.

Environmental finding	Implication for musculoskeletal experimental research
Fibers and fragments accounted for approximately 90% of classified particles	Experimental studies should not rely exclusively on spherical polymer beads
Fibers were approximately 2.51 times longer than fragments	Particle length should be treated as an experimental variable
Fibers had approximately 24.76 times the aspect ratio of fragments	Elongated and irregular morphologies should be evaluated separately
Polyester was predominantly fibrous, whereas polyethylene and polypropylene were more commonly fragmented	Polymer identity and particle geometry should be jointly controlled when selecting experimental particles
Dimensional and material information was incomplete	Experimental studies should use chemically confirmed particles and transparently report dimensions and morphology

### 4.4 Implications for environmental monitoring and future translational research

The findings of this study highlight several implications for environmental monitoring and future translational research. First, monitoring strategies should consider particle characteristics in addition to total abundance because morphology, size, and polymer composition may influence environmental behavior and biological relevance. Second, source-control strategies should consider major contributors identified in environmental profiles, including synthetic textile shedding, wastewater pathways, and plastic fragmentation processes (12, 13).

## 5 Limitations

Several limitations should be considered. The analysis used publicly available datasets generated through heterogeneous sampling, filtration, detection, and classification procedures. The two datasets also differed in observational unit: the San Francisco Bay inventory was particle-level, whereas the drinking-water compilation was record-level. They were therefore analyzed separately and cannot support direct quantitative comparison. Sampling effort and analytical sensitivity also differed across environmental matrices, meaning that pooled particle counts should not be interpreted as universal environmental-prevalence estimates.

Dimensional and material data were incomplete, and the availability of these variables may reflect source-specific subsampling procedures. Polymer occurrence within the characterized subset cannot therefore be extrapolated to all particles. The analyzed regions and sampling periods may not represent other geographic or temporal settings. Finally, the study did not measure human intake, internal dose, tissue accumulation, inflammatory biomarkers, or musculoskeletal outcomes. The findings should consequently be interpreted as environmental characterization and hypothesis-generating guidance for experimental design rather than as toxicological, epidemiological, or clinical evidence. Multivariate dispersion differed significantly among matrices; therefore, the PERMANOVA finding reflects a combination of differences in average morphology profiles and differences in within-matrix variability and should not be interpreted exclusively as a centroid effect.

## 6 Conclusion

This secondary analysis characterized microplastic morphology, dimensions, size distribution, and polymer composition across drinking-water and estuarine datasets. Fibers and fragments represented the predominant recorded morphologies, while particle dimensions and material assignments showed substantial heterogeneity. These findings indicate that environmental microplastics should not be treated as a uniform exposure defined solely by particle abundance or polymer identity.

The principal translational contribution of this study is the identification of environmentally observed combinations of particle morphology, dimensions, and material identity that may guide the selection of more representative particles for future cartilage-, synovium-, and bone-related experimental models. In particular, the predominance of fibers and fragments and the marked geometric differences between these morphologies indicate that future musculoskeletal studies should not rely exclusively on standardized spherical particles. Nevertheless, the present findings do not establish human exposure, tissue deposition, biological toxicity, clinical association, or a causal relationship between microplastics and musculoskeletal disease.

## Data Availability

Publicly available datasets were analyzed in this study. This data can be found here: the primary estuarine microplastic dataset analyzed in this study is publicly accessible as the file “2020-09-11_microparticledata.xlsx” from the San Francisco Bay Microplastics Project (San Francisco Estuary Institute and 5 Gyres Institute), hosted on the California Natural Resources Agency Open Data Portal at: https://data.cnra.ca.gov/dataset/microplastic-sf-bay/resource/4e6aaf6f-7055-484b-84f4-3a66df294f27. Supplementary drinking-water compilations and any derived datasets generated during the current study are available from the corresponding authors upon reasonable request.
